# Knowledge and attitudes towards dementia in a sample of medical residents from a university-hospital in São Paulo, Brazil

**DOI:** 10.1590/S1980-57642016DN10100007

**Published:** 2016

**Authors:** Alessandro Ferrari Jacinto, Paulo José Fortes Villas Boas, Vânia Ferreira de Sá Mayoral, Vanessa de Albuquerque Citero

**Affiliations:** 1 MD, PhD Associate Professor, Internal Medicine Department, Faculdade de Medicina de Botucatu - UNESP, Botucatu SP, Brazil; 2 MD Geriatrician, Internal Medicine Department, Faculdade de Medicina de Botucatu - UNESP, Botucatu SP, Brazil; 3 MD, PhD Psychiatrist, Psychiatry Department, Escola Paulista de Medicina - UNIFESP, São Paulo SP, Brazil

**Keywords:** health knowledge, attitudes, general practitioners, aged, dementia, conhecimento, atitude, clínicos gerais, idoso, demência

## Abstract

An estimated 61% of the 24.3 million people diagnosed with dementia worldwide live in underdeveloped countries, including Brazil, where a public healthcare system covers the majority of the population. This care is usually provided by General Practitioners (GP) and in Brazil many doctors recently graduated from medical school and residents of different medical specialties practice as GPs.

**Objective:**

The aim of this study was to describe the knowledge and attitudes about dementia in a sample of Brazilian medical residents from a university-hospital in São Paulo, Brazil.

**Methods:**

A total of 152 Brazilian medical residents participated in the study. Participants answered a "Knowledge Quiz" (KQ) and "Attitude Quiz" (AQ) about dementia issues, transculturally adapted for use in Brazilian physicians. A descriptive analysis of the correct answers on knowledge and of the attitude aspects was performed.

**Results:**

The medical residents showed poor knowledge (<50%) about dementia prevalence and incidence and a good knowledge on disease management and diagnosis. Participants tended to be optimistic about caring for demented patients.

**Conclusion:**

In this study, it is likely that the physicians' good knowledge about dementia issues is the reason for their optimism dealing with demented patients.

## RESUMO

Estima-se que 61% dos 24,3 milhões de pessoas diagnosticadas com demência no mundo vivem em países subdesenvolvidos, incluindo o Brasil, onde o sistema público de saúde (SUS) abrange a maioria da população. Os atendimentos médicos no SUS são realizados, habitualmente, por médicos generalistas. No Brasil, muitos médicos, logo após a graduação e residentes de diferentes especialidades médicas trabalham como médicos generalistas.

**Objetivo:**

O objetivo deste estudo foi descrever o conhecimento e as atitudes sobre demência em uma amostra de médicos residentes brasileiros de um hospital universitário em São Paulo, Brasil.

**Métodos:**

152 médicos residentes participaram do estudo respondendo a um instrumento sobre conhecimentos e atitudes sobre demências que havia sido adaptado transculturalmente para ser usado no Brasil. Uma análise descritiva das respostas corretas sobre conhecimentos e atitudes foi realizada.

**Resultados:**

Os médicos residentes mostraram pouco conhecimento (<50%) sobre a prevalência e incidência de demência e maior conhecimento em gerenciamento e diagnóstico. Os profissionais tenderam a ser otimistas sobre como cuidar de pacientes com demência.

**Conclusão:**

Neste estudo, provavelmente, o bom conhecimento sobre algumas questões de demência é a razão para esses médicos sentirem-se mais otimistas com o cuidado aos pacientes com demência.

## INTRODUCTION

The prevalence of dementia in Brazil has increased with the rapid aging of its population.^1^ An estimated 61% of the 24.3 million people diagnosed with dementia worldwide live in underdeveloped countries.^2^

Brazil has a public healthcare system that covers the majority of its population. Most older people in Brazil are cared for by General Practitioners (GP) who play an important role in the diagnosis and treatment of diseases prevalent in this population, such as dementia.^3^

However, GPs often overlook cognitive impairment (CI) in older people, especially during its early stages.^4^^-^ 7 The lack of CI detection by GPs has been studied in several countries but in Brazil only one study has published data on this issue.^8^ It is unclear whether early detection of dementia improves patient outcomes, although specialist medical societies recommend dementia screening as part of the global geriatric assessment.^9^^,^ 10

In Brazil, many doctors shortly after medical school graduation and residents of different medical specialties practice as general practitioners. This situation raises an important question: are these professionals sufficiently prepared to deal with demented older patients?

The aim of this study was to describe the knowledge and the attitudes about dementia in a sample of Brazilian residents from a university-hospital in São Paulo, Brazil.

## METHODS

This cross-sectional study was run in March 2014. The convenience sample comprised first-year medical residents from residence programs of the Escola Paulista de Medicina, Universidade Federal de São Paulo, Brazil (UNIFESP), a Brazilian public university. All 152 medical residents who attended the "Welcome session" for new residents were included in the study (comprising 32% of the total 471 first-year residents). 

The setting for this study was chosen for two reasons. First, many residents had recently graduated from medical school (less than three months), allowing the obtention of information about undergraduate teaching on dementia. Second, irrespective of residence programs, many residents were about to practice as GPs in the Brazilian public and private health systems for extra income, since their residence scholarships were insufficient for an adequate standard of living in the relatively expensive city of São Paulo. Many residents choose to work as GPs for a couple of years after graduating from Medical School, before joining residency programs later.^11^

 The physicians were assessed by an instrument that included a "Knowledge Quiz" and an "Attitude Quiz" about dementia issues previously used with UK general practitioners in the study by Turner et al.^12^ The Knowledge Quiz (KQ) has 14 tests with five possible multiple choice answers, including "I don't know", only one of which is correct. This questionnaire has three domains about dementia epidemiology, diagnosis and management. The Attitude Quiz (AQ) contains 10 statements conveying physicians' thoughts on the management of demented patients graded on a 5-point Likert-type scale (from "strongly agree" to "strongly disagree"). The KQ and AQ were translated, back-translated and culturally adapted for use in Brazil, where this process has been described elsewhere.^13^ The sample of residents also answered a semi-structured questionnaire regarding several aspects of dementia teaching during undergraduate medical training.

This project was approved by the Research Ethics Committee of the Escola Paulista de Medicina, Universidade Federal de São Paulo, Brazil.

**Statistical analysis.** Descriptive analyses were performed for all variables. 

## RESULTS

Physicians' mean age was 26 (±2.2) years. Forty-eight subjects (31.6%) were starting residence programs at the same medical school where they graduated (UNIFESP) while the remaining doctors were from 57 other Brazilian medical schools; 74 (48.1%) doctors had graduated within the last year. Sixty-one medical residents (40.8%) were from programs related to care of dementia patients (Neurology, Geriatrics, Psychiatry and Internal Medicine). The other medical residents were from surgery programs and other clinical subspecialties. 

Table 1 shows the results of the doctors' opinions on characteristics regarding dementia teaching. Of the 152 residents assessed, 79(52%) stated they received some training on dementia during medical school.

**Table 1.** Frequency of doctors' answers on dementia teaching during medical school

		**N**	**%**
There was dementia teaching during medical school	Yes Theoretical only Theoretical and practical	79 22 57	52.0 14.5 37.5
	No	69	40.1
	Missing	4	2.6
Attended extra-curricular course on dementia during medical school	Yes	7	4.6
	No	141	92.8
	Missing	4	2.6
**Total**		**152**	**100**

Table 2 shows the distribution of the correct answers on the Knowledge Quiz. With regard to epidemiology, diagnosis and management sub scales, 54.9, 58.7 and 76.3% of the sample, respectively, answered the questions correctly. On the epidemiology subscale, the two questions about prevalence and incidence aspects (Questions 1 and 2) had a low percentage of right answers but a good level of knowledge on etiology and risk factors of dementia (Questions 3 and 4) was exhibited by the medical residents (over 80% correct). The diagnostic subscale has 3 questions about diagnostic evaluation (Questions 5, 6 and 7) and a high percentage of correct answers on two of these was noted. The other four questions from this subscale concern symptoms of the disease, and knowledge about this was low. On the management subscale, the medical residents showed a high level of knowledge of antidementia drugs but had difficulty in treating dementia and depression as comorbidities.

**Table 2.** Distribution of correct answers on the Knowledge Quiz.

**Question**	**N (%)**
Epidemiology subscale	
A general practitioner with a list of 1,500-2,000 people can expect to have the following number of people with dementia on their list...	41 (27.2)
By 2021, the prevalence of dementia in the general population in Brazil is expected to...	36 (23.8)
One of the risk factors for the development of Alzheimer's disease is...	131 (86.8)
All of the following are potentially treatable etiologies of dementia except...	122 (81.9)
Diagnosis subscale	
A patient suspected of having dementia should be evaluated as soon as possible as...	134 (89.3)
Which of the following procedures is required to definitely confirm that symptoms are due to dementia?	56 (36.8)
Which of the following is not a necessary part of the initial evaluation of a patient with possible dementia?	139 (92.1)
Which of the following sometimes resembles dementia?	32 (21.1)
When a patient develops a sudden onset of confusion, disorientation, and inability to sustain attention, this presentation is most consistent with the diagnosis of ...	16 (10.5)
Which of the following is nearly always present in dementia?	5 (3.3)
Which of the following clinical findings best differentiates vascular dementia from Alzheimer's?	117 (77.0)
Management subscale	
The effect of anti-dementia drugs is to...	127 (83.6)
Which statement is true concerning the treatment of dementia patients who are depressed?	70 (46.1)
Which of the following best describes the functions of the Alzheimer's disease society?	151 (99.3)

Table 3 shows the distribution of the attitudes of the medical residents about treating demented patients. On 9 out of the 10 questions, attitudes toward dementia in older patients was optimistic: medical doctors felt able to help both patient and caregiver in primary care. By contrast, on the 5^th^ statement ("dementia is best diagnosed by specialist services"), 66% of the residents agreed that a specialized service is better than primary care for diagnosing dementia.

**Table 3.** Distribution of answers on the Attitude Quiz.

	**Totally agree % (N)**	**Agree % (N)**	**Not agree or disagree % (N)**	**Disagree % (N)**	**Totally disagree % (N)**
1. Much can be done to improve the quality of life of carers of people with dementia	74 (113)	24 (37)	1 (1)	1 (1)	0
2. Families would rather be told about their relative's dementia as soon as possible	33 (50)	41 (62)	24 (36)	3 (4)	0
3. Much can be done to improve the quality of life of people with dementia	63 (95)	35 (53)	3 (4)	0	0
4. Providing diagnosis is usually more helpful than harmful	38 (57)	46 (70)	15 (22)	2 (3)	0
5. Dementia is best diagnosed by specialist services	18 (28)	48 (73)	18 (28)	14 (21)	1 (2)
6. Patients with dementia can be a drain on resources with little positive outcome	5 (8)	32 (49)	29 (44)	30 (45)	3 (5)
7. It is better to talk to the patient in euphemistic terms	0	3 (4)	22 (34)	54 (82)	21 (32)
8. Managing dementia is more often frustrating than rewarding	1 (2)	16 (25)	32 (49)	42 (64)	8 (12)
9. There is little point in referring families to services as they do not want to use them	1 (1)	18 (28)	22 (33)	50 (76)	9 (14)
10. The primary care team has a very limited role to play in the care of people with dementia	1 (2)	5 (7)	8 (12)	40 (61)	46 (70)

## DISCUSSION

Although designed to assess attitudes toward and knowledge of dementia in General Practitioners from the United Kingdom, these instruments seemed adequate for the same purpose among Brazilian physicians, providing a basic assessment of the topics. 

In Brazil, the medical doctors were more confident managing and diagnosing dementia than understanding the epidemiologic issues about the disease. Considering the setting of a primary care delivery service, the medical residents showed good performance for patient care and good knowledge. It seems that recently graduated physicians are being adequately prepared to deal with this important condition. The comorbidity between depression and dementia is a prevalent aspect requiring more emphasis during medical training.

It is likely that the good knowledge about dementia is the reason for medical doctors' optimism dealing with demented patients in primary care. Considering that 60% of the study sample comprised medical residents from programs not related to the care of dementia patients, these optimistic attitudes may reflect a good foundation on the issue during medical school.

In the United Kingdom, akin to Brazil, General Practitioners are responsible for treating the majority of people diagnosed with dementia, representing a prime example of a public health care system strongly centered on primary care.^14^ The UK National Dementia Strategy has highlighted gaps in the knowledge and skills of primary care professionals regarding the care of patients with dementia.^15^ Tullo et al., after applying a questionnaire about teaching and learning on dementia to 23 of the UK's 31 medical schools, found a lack of consensus among medical schools on how to teach dementia.^16^

In Brazil, as is the case for most countries worldwide, there is scant information on the education given to medical students about dementia during medical school.^17^^,^ 18 One report from a survey applied in a public medical school showed that 61.7% of the medical students received no training in Geriatrics.^19^ Another study showed that 42% of Brazilian medical schools provided teaching in Geriatrics.^20^

This study has several limitations. Since only one sample of residents from a single university-hospital was analyzed, it was not possible to glean a clear picture of dementia teaching in the numerous Brazilian medical schools. Considering the differences found between Brazilian and British doctors' attitudes, no further conclusions can be drawn since these two populations differ in many aspects, such as graduation time and age, besides the type of medical education received during their careers. 

In conclusion, this study revealed important aspects regarding dementia issues and can serve as a basis for further discussions about dementia teaching in Brazil.
